# *Xanthichthys greenei*, a new species of triggerfish (Balistidae) from the Line Islands

**DOI:** 10.3897/BDJ.1.e994

**Published:** 2013-09-16

**Authors:** Richard L. Pyle, John L. Earle

**Affiliations:** † Bishop Museum, Honolulu, United States of America

**Keywords:** Balistidae, *Xanthichthys*, new species, Mesophotic Coral Ecosystem, Line Islands

## Abstract

*Xanthichthys greenei* sp. n. is described from six specimens, 97-154 mm standard length (SL) collected from mesophotic coral ecosystems (90-100 m) at Kiritimati (Christmas Island), Line Islands, part of the Republic of Kiribati in the Central Pacific. Of the six species of *Xanthichthys*, it is most similar to the Atlantic *Xanthichthys ringens* and the Indo-West Pacific *Xanthichthys lineopunctatus*, sharing with these species the character of three pigmented cheek grooves. It is distinctive in its low body scale row count (33-35, other *Xanthichthys* species with 39 or more), small size (maximum SL 154 mm, other species over 225 mm), and color pattern of scattered dark spots sub-dorsally and no other spots or lines on body.

## Introduction

In their revision of the triggerfish genus *Xanthichthys* Kaup in Richardson (1856), Randall et al. (1978) recognized five distinct species within this genus: the Atlantic *Xanthichthys ringens* (Linnaeus 1758); the Indo-Pacific *Xanthichthys lineopunctatus* (Hollard 1854), *Xanthichthys caeruleolineatus*Randall et al. 1978, and *Xanthichthys auromarginatus* (Bennett 1832); and the Pacific *Xanthichthys mento* (Jordan and Gilbert 1882). In July 2005, the authors, accompanied by Brian D. Greene, conducted a series of deep exploratory dives using rebreathers to assess the deep reef (mesophotic) fish fauna off the north and west shores of Kiritimati (Christmas Island, Line Islands). Among several undescribed species of fishes observed or collected below 80-85 m off the Captain Cook Hotel on the north shore of Kiritimati were six specimens of a new species of *Xanthichthys*. Numerous other individuals of this new triggerfish were observed, and the collected specimens are representative of the size range of observed individuals. All individuals of this new species were encountered at depths of 90-100 m, below a strong thermocline at a variable depth of 80-85 m (the water temperature was approximately 25-28°C above the thermocline, and approximately 20-22°C below). The habitat consisted of a slightly downward sloping hard substratum with patches of rocks approximately 15-30 cm in size, with occasional small holes providing habitat and cover for fishes. At 100 m the slope transitioned to a vertical wall approximately 4 m high, with another thermocline and a steep downward slope below this wall in very cold water (approximately 10-14°C). No *Xanthichthys* were observed associated with or below this wall, or above the 80-85 meter thermocline. However within these depth parameters, a narrow band of approximately 30 m width, the *Xanthichthys* species appeared to be common.

## Materials and methods

Deep dives were made using Cis-Lunar Mk-5P mixed-gas, closed-circuit rebreathers. Specimens were collected by hand and by hand net.

Counts and measurements follow the methods described by Randall et al. (1978) in their revision of the genus. Body-scale rows were counted from the upper end of the gill opening to the caudal base. These counts include the diagonal rows of small scales just behind the gill opening. Head-scale rows were counted from just posterior to the corner of the mouth to the upper end of the gill opening. This count includes small scales at either end. Because scales on the head were not in perfect alignment and because of intercalated rows, precise and consistent head-scale rows were difficult to obtain. We follow Randall et al. (1978) in excluding gill raker counts, both because this count is inconclusive for differentiating species within the genus, and because of the necessity to damage specimens to access gill rakers.

Standard length (SL) was measured from the tip of the snout to the caudal base. Depth of body, width of body, head length, snout length, tip of snout to origins of first dorsal and anal fins, and length of second dorsal-fin and anal-fin bases, eye diameter, interorbital width, length of gill opening, depth of caudal peduncle, length of first dorsal spine, length of longest dorsal soft ray, length of longest anal ray, interdorsal space, length of caudal fin, caudal concavity, and length of pectoral fin are expressed as percent of SL. Head length was measured from the tip of the snout to the upper end of the gill opening.

Maximum depth measurement may be variable due to the degree of extension of the pelvic flap when the individual specimen was preserved. Width of the body is the maximum width. Depth of the caudal peduncle is the least depth. Eye diameter is the maximum fleshy diameter. Interorbital width is the least bony width. Interdorsal space is the distance from the posterior edge of the first dorsal spine at its base to the origin of the first ray of the second dorsal fin (excluding the basal scaly sheath). Measurement of the longest ray of the second dorsal and anal fins exclude the basal scaly sheath. Bases of the second dorsal and anal fins were measured from the anterior margin of the first ray at the upper end of the basal scaly sheath to the posterior edge of the last ray. Caudal concavity is the horizontal distance between the distal tips of the longest and shortest caudal rays.

Tissue samples intended for DNA sequencing were removed from collected specimens prior to formalin fixation in the field, but these tissue samples were subsequently lost in transit. Description of this species was postponed for eight years in anticipation of obtaining additional specimens from Kiritimati or other nearby localities, but following exploration of similar habitats in French Polynesia, the Cook Islands, and Johnston Atoll without observing this species, we have elected to describe this new species without molecular analysis.

The holotype has been deposited at the Bernice Pauahi Bishop Museum fish collection, Honolulu (BPBM); and paratypes have been deposited at BPBM, the California Academy of Sciences fish collection, San Francisco (CAS), and the U.S. National Museum of Natural History, Washington, D.C. (USNM).

## Taxon treatments

### Xanthichthys greenei

Pyle and Earle sp. n.

urn:lsid:zoobank.org:act:28D73F00-F8E0-4997-9E02-FF70B2571CE5

#### Materials

- **Type status:** Holotype. **Occurrence:** catalogNumber: 40262; recordedBy: Brian D. Greene; individualCount: 1; sex: male; preparations: whole animal (55% Isopropanol); **Taxon:** taxonID: 28D73F00-F8E0-4997-9E02-FF70B2571CE5; scientificNameID: 28D73F00-F8E0-4997-9E02-FF70B2571CE5; acceptedNameUsageID: 28D73F00-F8E0-4997-9E02-FF70B2571CE5; parentNameUsageID: EDB706F7-A7E1-4763-AB97-ECB71151CDBD; originalNameUsageID: 28D73F00-F8E0-4997-9E02-FF70B2571CE5; nameAccordingToID: A64FB7C8-41F7-4DAE-B538-FE3BEA1E3DFF; namePublishedInID: A64FB7C8-41F7-4DAE-B538-FE3BEA1E3DFF; scientificName: Xanthichthys greenei; acceptedNameUsage: Xanthichthys greenei Pyle & Earle; parentNameUsage: Xanthichthys Kaup in Richardson, 1856; originalNameUsage: Xanthichthys greenei Pyle & Earle; nameAccordingTo: Pyle, Richard L. & John L. Earle. In Press. Xanthichthys greenei, a new species of triggerfish (Balistidae) from the Line Islands. Biodiversity Data Journal.; namePublishedIn: Pyle, Richard L. & John L. Earle. In Press. Xanthichthys greenei, a new species of triggerfish (Balistidae) from the Line Islands. Biodiversity Data Journal.; higherClassification: Animalia; Deuterostomia; Chordata; Craniata; Gnathostomata; Actinopterygii; Tetraodontiformes; Tetraodontoidei; Balistoidea; Balistidae; Xanthichthys; kingdom: Animalia; phylum: Deuterostomia; class: Actinopterygii; order: Tetraodontiformes; family: Balistidae; genus: Xanthichthys; specificEpithet: greenei; taxonRank: species; scientificNameAuthorship: Pyle and Earle; vernacularName: Greene's Triggerfish; nomenclaturalCode: ICZN; **Location:** higherGeography: Pacific Ocean; Central Pacific; waterBody: Pacific Ocean; islandGroup: Line Islands; island: Kiritimati Atoll (Christmas Island); country: Kiribati; countryCode: KI; locality: directly off fuel storage tanks near London; outside wreck of freighter ship; verbatimLocality: Line Islands; Kiritimati Atoll; directly off fuel storage tanks near London; outside wreck of freighter ship. 300 feet.; verbatimDepth: 300 feet; minimumDepthInMeters: 90; maximumDepthInMeters: 90; verbatimLatitude: 1°59'24.7884"N; verbatimLongitude: 157°29'10.32"W; verbatimCoordinateSystem: degrees minutes seconds; verbatimSRS: WGS84; decimalLatitude: 1.990219°; decimalLongitude: -157.486200°; geodeticDatum: WGS84; coordinateUncertaintyInMeters: 500; **Identification:** identifiedBy: Richard L. Pyle; dateIdentified: 2013-08-23; **Event:** samplingProtocol: Caught by hand; eventDate: 2005-07-22; year: 2005; month: 7; day: 22; verbatimEventDate: 22 July 2005; habitat: rubble on silty sand slope; **Record Level:** collectionID: urn:lsid:biocol.org:col:1001; institutionCode: BPBM; collectionCode: Fish; basisOfRecord: PreservedSpecimen
- **Type status:** Paratype. **Occurrence:** catalogNumber: 40228; recordedBy: Brian D. Greene; individualCount: 1; preparations: whole animal (55% Isopropanol); **Taxon:** taxonID: 28D73F00-F8E0-4997-9E02-FF70B2571CE5; scientificNameID: 28D73F00-F8E0-4997-9E02-FF70B2571CE5; acceptedNameUsageID: 28D73F00-F8E0-4997-9E02-FF70B2571CE5; parentNameUsageID: EDB706F7-A7E1-4763-AB97-ECB71151CDBD; originalNameUsageID: 28D73F00-F8E0-4997-9E02-FF70B2571CE5; nameAccordingToID: A64FB7C8-41F7-4DAE-B538-FE3BEA1E3DFF; namePublishedInID: A64FB7C8-41F7-4DAE-B538-FE3BEA1E3DFF; scientificName: Xanthichthys greenei; acceptedNameUsage: Xanthichthys greenei Pyle & Earle; parentNameUsage: Xanthichthys Kaup in Richardson, 1856; originalNameUsage: Xanthichthys greenei Pyle & Earle; nameAccordingTo: Pyle, Richard L. & John L. Earle. 2013. Xanthichthys greenei, a new species of triggerfish (Balistidae) from the Line Islands. Biodiversity Data Journal.; namePublishedIn: Pyle, Richard L. & John L. Earle. 2013. Xanthichthys greenei, a new species of triggerfish (Balistidae) from the Line Islands. Biodiversity Data Journal.; higherClassification: Animalia; Deuterostomia; Craniata; Gnathostomata; Actinopterygii; Tetraodontiformes; Tetraodontoidei; Balistoidea; Balistidae; Xanthichthys; kingdom: Animalia; phylum: Deuterostomia; class: Actinopterygii; order: Tetraodontiformes; family: Balistidae; genus: Xanthichthys; specificEpithet: greenei; taxonRank: species; scientificNameAuthorship: Pyle and Earle; vernacularName: Greene's Triggerfish; nomenclaturalCode: ICZN; **Location:** higherGeography: Pacific Ocean; Central Pacific; waterBody: Pacific Ocean; islandGroup: Line Islands; island: Kiritimati Atoll (Christmas Island); country: Kiribati; countryCode: KI; locality: N of Poland; verbatimLocality: Line Islands; Kiritimati Atoll; N of Poland. 320 feet.; verbatimDepth: 320 feet; minimumDepthInMeters: 96; maximumDepthInMeters: 96; verbatimLatitude: 1°53.282'N; verbatimLongitude: 157°33.287'W; verbatimCoordinateSystem: degrees decimal minutes; verbatimSRS: WGS84; decimalLatitude: 1.888033°; decimalLongitude: -157.554783°; geodeticDatum: WGS84; coordinateUncertaintyInMeters: 300; **Identification:** identifiedBy: Richard L. Pyle; dateIdentified: 2013-08-23; **Event:** samplingProtocol: Caught by hand; eventDate: 2005-07-19; year: 2005; month: 7; day: 19; verbatimEventDate: 19 July 2005; habitat: crest of deep drop-off, below deep sand & rubble slope; **Record Level:** collectionID: urn:lsid:biocol.org:col:1001; institutionCode: BPBM; collectionCode: Fish; basisOfRecord: PreservedSpecimen
- **Type status:** Paratype. **Occurrence:** catalogNumber: 40342; recordedBy: Brian D. Greene; individualCount: 2; preparations: whole animal (55% Isopropanol); **Taxon:** taxonID: 28D73F00-F8E0-4997-9E02-FF70B2571CE5; scientificNameID: 28D73F00-F8E0-4997-9E02-FF70B2571CE5; acceptedNameUsageID: 28D73F00-F8E0-4997-9E02-FF70B2571CE5; parentNameUsageID: EDB706F7-A7E1-4763-AB97-ECB71151CDBD; originalNameUsageID: 28D73F00-F8E0-4997-9E02-FF70B2571CE5; nameAccordingToID: A64FB7C8-41F7-4DAE-B538-FE3BEA1E3DFF; namePublishedInID: A64FB7C8-41F7-4DAE-B538-FE3BEA1E3DFF; scientificName: Xanthichthys greenei; acceptedNameUsage: Xanthichthys greenei Pyle & Earle; parentNameUsage: Xanthichthys Kaup in Richardson, 1856; originalNameUsage: Xanthichthys greenei Pyle & Earle; nameAccordingTo: Pyle, Richard L. & John L. Earle. 2013. Xanthichthys greenei, a new species of triggerfish (Balistidae) from the Line Islands. Biodiversity Data Journal.; namePublishedIn: Pyle, Richard L. & John L. Earle. 2013. Xanthichthys greenei, a new species of triggerfish (Balistidae) from the Line Islands. Biodiversity Data Journal.; higherClassification: Animalia; Deuterostomia; Craniata; Gnathostomata; Actinopterygii; Tetraodontiformes; Tetraodontoidei; Balistoidea; Balistidae; Xanthichthys; kingdom: Animalia; phylum: Deuterostomia; class: Actinopterygii; order: Tetraodontiformes; family: Balistidae; genus: Xanthichthys; specificEpithet: greenei; taxonRank: species; scientificNameAuthorship: Pyle and Earle; vernacularName: Greene's Triggerfish; nomenclaturalCode: ICZN; **Location:** higherGeography: Pacific Ocean; Central Pacific; waterBody: Pacific Ocean; islandGroup: Line Islands; island: Kiritimati Atoll (Christmas Island); country: Kiribati; countryCode: KI; locality: off New Village; verbatimLocality: Line Islands; Kiritimati Atoll; off New Village; verbatimDepth: 300-330 feet; minimumDepthInMeters: 90; maximumDepthInMeters: 100; verbatimLatitude: 2°0'57.8196"N; verbatimLongitude: 157°29'45.2652"W; verbatimCoordinateSystem: degrees decimal minutes; verbatimSRS: WGS84; decimalLatitude: 2.016061°; decimalLongitude: -157.495907°; geodeticDatum: WGS84; coordinateUncertaintyInMeters: 300; **Identification:** identifiedBy: Richard L. Pyle; dateIdentified: 2013-08-23; **Event:** samplingProtocol: Quinaldine and hand net; eventDate: 2005-07-28; year: 2005; month: 7; day: 28; verbatimEventDate: 28 July 2005; habitat: rubble patch at crest of deep drop-off; **Record Level:** collectionID: urn:lsid:biocol.org:col:1001; institutionCode: BPBM; collectionCode: Fish; basisOfRecord: PreservedSpecimen
- **Type status:** Paratype. **Occurrence:** catalogNumber: 410688; recordedBy: Brian D. Greene; individualCount: 1; preparations: whole animal (55% Isopropanol); otherCatalogNumbers: BPBM 40342; **Taxon:** taxonID: 28D73F00-F8E0-4997-9E02-FF70B2571CE5; scientificNameID: 28D73F00-F8E0-4997-9E02-FF70B2571CE5; acceptedNameUsageID: 28D73F00-F8E0-4997-9E02-FF70B2571CE5; parentNameUsageID: EDB706F7-A7E1-4763-AB97-ECB71151CDBD; originalNameUsageID: 28D73F00-F8E0-4997-9E02-FF70B2571CE5; nameAccordingToID: A64FB7C8-41F7-4DAE-B538-FE3BEA1E3DFF; namePublishedInID: A64FB7C8-41F7-4DAE-B538-FE3BEA1E3DFF; scientificName: Xanthichthys greenei; acceptedNameUsage: Xanthichthys greenei Pyle & Earle; parentNameUsage: Xanthichthys Kaup in Richardson, 1856; originalNameUsage: Xanthichthys greenei Pyle & Earle; nameAccordingTo: Pyle, Richard L. & John L. Earle. 2013. Xanthichthys greenei, a new species of triggerfish (Balistidae) from the Line Islands. Biodiversity Data Journal.; namePublishedIn: Pyle, Richard L. & John L. Earle. 2013. Xanthichthys greenei, a new species of triggerfish (Balistidae) from the Line Islands. Biodiversity Data Journal.; higherClassification: Animalia; Deuterostomia; Craniata; Gnathostomata; Actinopterygii; Tetraodontiformes; Tetraodontoidei; Balistoidea; Balistidae; Xanthichthys; kingdom: Animalia; phylum: Deuterostomia; class: Actinopterygii; order: Tetraodontiformes; family: Balistidae; genus: Xanthichthys; specificEpithet: greenei; taxonRank: species; scientificNameAuthorship: Pyle and Earle; vernacularName: Greene's Triggerfish; nomenclaturalCode: ICZN; **Location:** higherGeography: Pacific Ocean; Central Pacific; waterBody: Pacific Ocean; islandGroup: Line Islands; island: Kiritimati Atoll (Christmas Island); country: Kiribati; countryCode: KI; locality: off New Village; verbatimLocality: Line Islands; Kiritimati Atoll; off New Village; verbatimDepth: 300-330 feet; minimumDepthInMeters: 90; maximumDepthInMeters: 100; verbatimLatitude: 2°0'57.8196"N; verbatimLongitude: 157°29'45.2652"W; verbatimCoordinateSystem: degrees decimal minutes; verbatimSRS: WGS84; decimalLatitude: 2.016061°; decimalLongitude: -157.495907°; geodeticDatum: WGS84; coordinateUncertaintyInMeters: 300; **Identification:** identifiedBy: Richard L. Pyle; dateIdentified: 2013-08-23; **Event:** samplingProtocol: Quinaldine and hand net; eventDate: 2005-07-28; year: 2005; month: 7; day: 28; verbatimEventDate: 28 July 2005; habitat: rubble patch at crest of deep drop-off; **Record Level:** collectionID: urn:lsid:biocol.org:col:1002; institutionCode: USNM; collectionCode: Fish; basisOfRecord: PreservedSpecimen
- **Type status:** Paratype. **Occurrence:** catalogNumber: 236257; recordedBy: Brian D. Greene; individualCount: 1; preparations: whole animal (55% Isopropanol); otherCatalogNumbers: BPBM 40342; **Taxon:** taxonID: 28D73F00-F8E0-4997-9E02-FF70B2571CE5; scientificNameID: 28D73F00-F8E0-4997-9E02-FF70B2571CE5; acceptedNameUsageID: 28D73F00-F8E0-4997-9E02-FF70B2571CE5; parentNameUsageID: EDB706F7-A7E1-4763-AB97-ECB71151CDBD; originalNameUsageID: 28D73F00-F8E0-4997-9E02-FF70B2571CE5; nameAccordingToID: A64FB7C8-41F7-4DAE-B538-FE3BEA1E3DFF; namePublishedInID: A64FB7C8-41F7-4DAE-B538-FE3BEA1E3DFF; scientificName: Xanthichthys greenei; acceptedNameUsage: Xanthichthys greenei Pyle & Earle; parentNameUsage: Xanthichthys Kaup in Richardson, 1856; originalNameUsage: Xanthichthys greenei Pyle & Earle; nameAccordingTo: Pyle, Richard L. & John L. Earle. 2013. Xanthichthys greenei, a new species of triggerfish (Balistidae) from the Line Islands. Biodiversity Data Journal.; namePublishedIn: Pyle, Richard L. & John L. Earle. 2013. Xanthichthys greenei, a new species of triggerfish (Balistidae) from the Line Islands. Biodiversity Data Journal.; higherClassification: Animalia; Deuterostomia; Craniata; Gnathostomata; Actinopterygii; Tetraodontiformes; Tetraodontoidei; Balistoidea; Balistidae; Xanthichthys; kingdom: Animalia; phylum: Deuterostomia; class: Actinopterygii; order: Tetraodontiformes; family: Balistidae; genus: Xanthichthys; specificEpithet: greenei; taxonRank: species; scientificNameAuthorship: Pyle and Earle; vernacularName: Greene's Triggerfish; nomenclaturalCode: ICZN; **Location:** higherGeography: Pacific Ocean; Central Pacific; waterBody: Pacific Ocean; islandGroup: Line Islands; island: Kiritimati Atoll (Christmas Island); country: Kiribati; countryCode: KI; locality: off New Village; verbatimLocality: Line Islands; Kiritimati Atoll; off New Village; verbatimDepth: 300-330 feet; minimumDepthInMeters: 90; maximumDepthInMeters: 100; verbatimLatitude: 2°0'57.8196"N; verbatimLongitude: 157°29'45.2652"W; verbatimCoordinateSystem: degrees decimal minutes; verbatimSRS: WGS84; decimalLatitude: 2.016061°; decimalLongitude: -157.495907°; geodeticDatum: WGS84; coordinateUncertaintyInMeters: 300; **Identification:** identifiedBy: Richard L. Pyle; dateIdentified: 2013-08-23; **Event:** samplingProtocol: Quinaldine and hand net; eventDate: 2005-07-28; year: 2005; month: 7; day: 28; verbatimEventDate: 28 July 2005; habitat: rubble patch at crest of deep drop-off; **Record Level:** collectionID: urn:lsid:biocol.org:col:1003; institutionCode: CAS; collectionCode: Fish; basisOfRecord: PreservedSpecimen

#### Description

Proportional measurements expressed as a percentage of standard length (SL). Data in parentheses apply to paratypes, when different from the holotype (see also Table 1).

Dorsal soft rays 29; anal rays 25; pectoral rays 13 (rarely 14); body scale rows 34 (33-35); head scale rows 18 (17-18); vertebrae 18, gill rakers 36 (holotype only).

Greatest depth of body 49 (45-50), depth at origin of anal fin 41 (42-43), width of body 29 (19-20), head length 32 (31-33), snout length 21 (20-21), snout to origin of first dorsal fin 33 (32-35), snout to origin of anal fin 69 (67-69), base of second dorsal fin 36 (35-37), base of anal fin 30 (30-32).

Eye diameter 6.5 (7.2-8.8), interorbital width 12 (12-13), length of gill opening 10 (9-10), depth of caudal peduncle 8.4 (8.4-9.0), length of first dorsal spine 14 (13-17), length of longest dorsal soft ray 19 (19-22), length of longest anal ray 18 (16-19), interdorsal space 25 (23-25), length of caudal fin 26 (21-26), caudal concavity 4.5 (2.1-5.8), length of pectoral fin 11 (12-14).

Dorsal and ventral profiles of head strongly convex; three longitudinal slightly diagonal darkly pigmented grooves on head following border of scale rows, running from behind and below corner of mouth, converging slightly as they pass posteriorly, and nearly reaching gill opening and upper pectoral base; a groove running anteriorly and diagonally downward from front of eye, extending approximately half the distance to tip of snout; deep medial groove behind first dorsal fin extending approximately half distance from last spine to origin second dorsal; pelvic flap not developed, pelvic terminus barely movable, small, slightly over one half orbit diameter.

**Figure 1.**
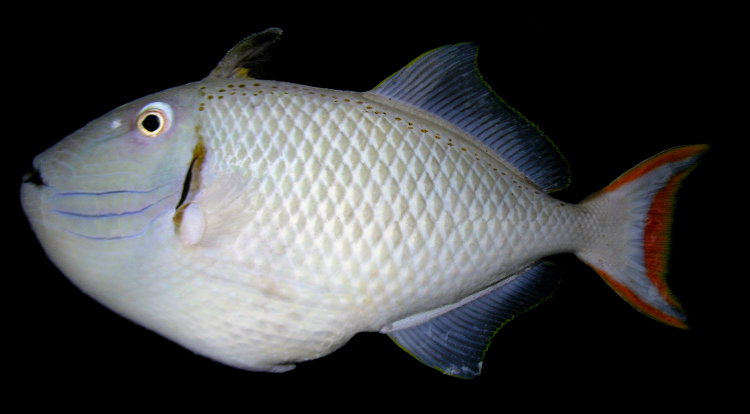
Holotype of *Xanthichthys greenei* (BPBM 40262), from Kiritimati (Christmas Island), Line Islands. Photo: R.L. Pyle.

Color in life as in Figs 1, 2; pearly gray, some paratypes with light brownish cast, slightly whiter ventrally on body, faint light blueish cast to head; irregular honey-brown spots sub-dorsally on body in several irregular rows commencing anteriorly at the origin of the first dorsal spine, becoming an irregular row prior to the origin of the second dorsal and ending posteriorly with a few scattered spots on the caudal peduncle; honey-brown diffuse bar below the anterior sub-dorsal spots and immediately posterior to gill opening, running from mid-orbit to slightly below mid-anterior pectoral base, opercular membrane darkly pigmented; three cheek grooves pigmented pale blue, dark on some paratypes, a weak groove above the three pigmented grooves faintly pigmented on some paratypes, groove extending anteriorly and diagonally downward from eye with faint blue pigment on some specimens; dark brown line below lower lip, thickening and extending diffusely beyond corner of mouth; first dorsal fin light tan brown becoming dark distally, first dorsal fin membrane basally tan, dark brown distally, second dorsal and anal fins with light brown rays, light blueish membranes; caudal fin with reddish upper and lower margins, a broad reddish crescent posteriorly in fin not extending to upper and lower margins, a light blueish area forming a diffuse crescent in the center of the fin; pectoral fin not pigmented. When viewed alive underwater the overall color appears light blue-gray.

Color in alcohol tan, paler ventrally; pale brown spots dorsally from origin of dorsal fin to caudal peduncle, more spots anteriorly; grooves in cheek brown; opercular membrane brown, faint brown bar posterior to gill opening from mid-orbit to mid pectoral base; brown line below lower lip extending diffusely beyond corner of mouth; anterior nostril in a small white spot; first dorsal spine light brown, darker distally on some specimens, membrane of first dorsal fin brown, darker posteriorly; second dorsal and anal fins pale with light brown rays; caudal fin pale yellow; pectoral fins translucent.

#### Diagnosis

Dorsal rays III, 29; anal rays 25; pectoral rays 13–14 (usually 13); body scale rows 33-35; head scale rows 17–18; gill rakers 36; body depth 2–2.22 in SL; color in life pearly gray with honey-brown spots sub-dorsally on body in several irregular rows between the first dorsal spine and the caudal peduncle; opercular membrane darkly pigmented with three cheek grooves pigmented pale blue; first dorsal fin light tan brown becoming dark distally, first dorsal fin membrane basally tan, dark brown distally, second dorsal and anal fins with light brown rays, light blueish membranes; caudal fin with reddish upper and lower margins, a broad reddish crescent posteriorly in fin not extending to upper and lower margins.

#### Etymology

Named for Brian D. Greene, a member of the deep diving team that discovered species, in recognition of his efforts to collect the type specimens.

#### Ecology

Relatively abundant within its depth range and habitat (coral rubble and holes adjacent to deeper drop-offs, below a thermocline) at several localities on the west and north coasts of Kiritimati (Christmas Island). Always observed near the reef substratum, where it would retreat to shelter when approached.

#### Distribution

Currently known only from Kiritimati (Christmas Island) in the Line Islands. Further exploration of mesophotic coral ecosystems in the tropical Pacific may extend the known range of this species, although it was not observed during exploratory dives within the mesophotic depth range in the Hawaiian Islands, Johnston Atoll, Caroline Islands, Cook Islands, French Polynesia, or elsewhere in the Pacific.

## Identification Keys

### Revised Key to the Species of *Xanthichthys*

**Table:** 

1	Cheek with three prominent slightly diagonal dark brown or blue grooves (a shorter narrower groove above and below the main three sometimes dark on *Xanthichthys lineopunctatus*), extending from just behind and below corner of mouth nearly to gill opening; body with dark longitudinal lines and/or spots	2
–	Cheek with five or six grooves (darkly pigmentd only on *Xanthichthys mento*; poorly developed on *Xanthichthys auromarginatus*); body without dark longitudinal lines and spots except males of *Xanthichthys mento* with a small light blue spot in scale centers that may be brown in preservative (in addition, a concentration of dark pigment corners of the scales of some specimens of *Xanthichthys mento*, giving a dark-spotted effect)	4
2	Lines or spots on body scales; scale rows between upper end of gill opening and caudal base 39-50	3
–	Lines or spots on body restricted to row of brown spots dorsally; scale rows between upper end of gill opening and caudal base 33-35 (Line Islands)	*Xanthichthys greenei*, new species
3	Body with a single round to longitudinally elliptical dark brown spot at corner of each scale; scales between upper end of gill opening and caudal fin base 39 to 44 (tropical Atlantic)	*Xanthichthys ringens*
–	Upper half of body with longitudinal dark brown lines following scale rows, lower half with a dark brown dash or spot at corner of each scale; scales between upper end of gill opening and caudal fin base 44 to 50 (Indian Ocean, Australia, and Japan)	*Xanthichthys lineopunctatus*
4	Depth of body at anal fin origin 2.45 to 2.68 in standard length; scales of body with a prominent median elevation, forming longitudinal ridges (better developed posteriorly) (Indo-Pacific)	*Xanthichthys auromarginatus*
–	Depth of body at anal fin origin 2.7 to 3.43 in standard length; scales of body with only slight median ridges posteriorily on body	5
5	Dorsal soft rays 29 to 32; anal soft rays 26 to 29; no longitudinal blue line on body (eastern, central and western Pacific)	*Xanthichthys mento*
–	Dorsal soft rays 26 to 28; anal soft rays 23 to 25; an irregular longitudinal blue line on body from pectoral axil to upper side of caudal peduncle (Indo-Pacific)	*Xanthichthys caeruleolineatus*

Based on the key included in Randall et al. (1978).

## Discussion

Table 2 compares *Xanthichthys* species using counts from Randall et al. (1978) and counts we have made for *Xanthichthys greenei*. The six known *Xanthichthys* species can be separated into two groups according to the number of cheek grooves, 3 or 5-6. *Xanthichthys ringens*, *Xanthichthys lineopunctatus* and *Xanthichthys greenei* all have three pigmented cheek grooves and share similarities in color pattern. Color of the caudal fin, soft dorsal and anal fin is nearly identical in these three species. However in *Xanthichthys greenei* the body spots of *Xanthichthys ringens* and the spots and lines of *Xanthichthys lineopunctatus* are reduced to a few spots dorsally. Another difference is that the base of the second dorsal and anal fins of *Xanthichthys greenei* is not darkly pigmented as it is on the other two species. *Xanthichthys greenei* can also be separated from these two species and from all other *Xanthichthys* species by its low body scale row count, which has no overlap with any other *Xanthichthys*. Moreover, it appears to be a small species, and the size of the holotype at 154 mm SL, as large as any we observed underwater, is considerably smaller than the maximum size recorded for the next smallest species (*Xanthichthys auromarginatus* at 227 mm).

While *Xanthichthys greenei* has only been recorded from Kiritimati, we would expect it to be present in suitable habitat throughout the northern Line Islands, possibly the southern Line Islands (but the junior author has noted faunal differences between the northern and southern Line Islands), and perhaps the Phoenix Islands. Although the habitat of *Xanthichthys greenei* is below normal scuba diving depths and the species could be missed by divers, Balistidae can be readily caught by hook and line from deep water by local fishermen. Since this species has not been recorded from French Polynesia or other heavily fished areas in the Central Pacific (despite comparable observations at similar depths and habitats using mixed-gas rebreathers), we are inclined to doubt its presence there.

## Supplementary Material

XML Treatment for Xanthichthys greenei

## Figures and Tables

**Figure 2a.**
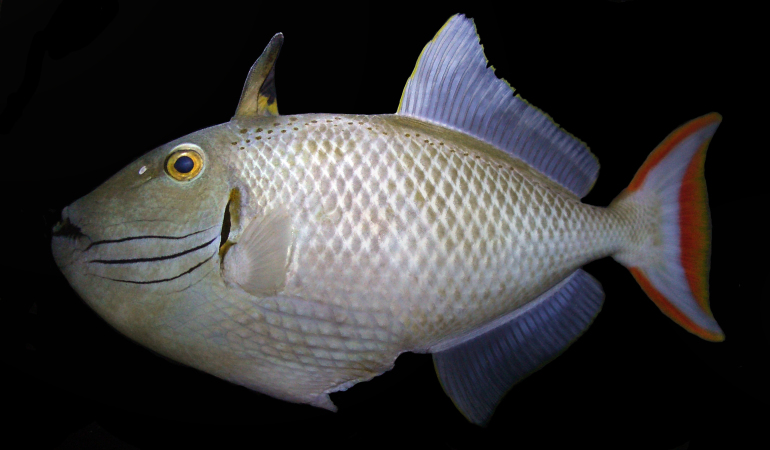


**Figure 2b.**
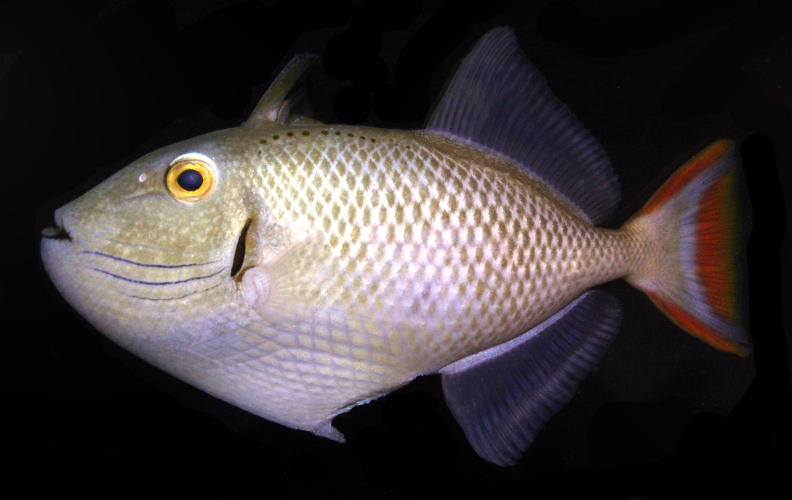


**Figure 2c.**
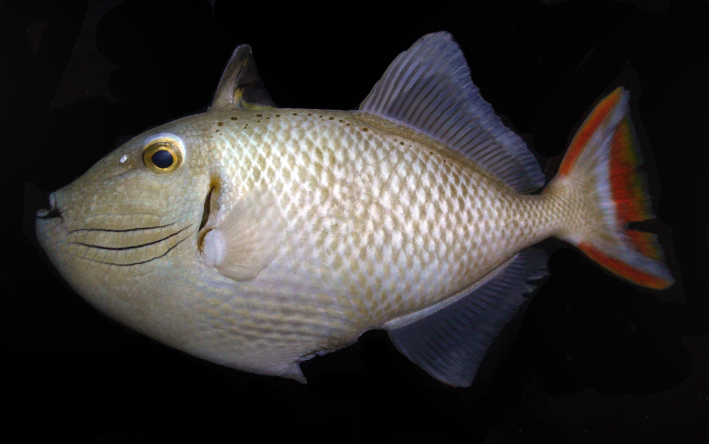


**Figure 2d.**
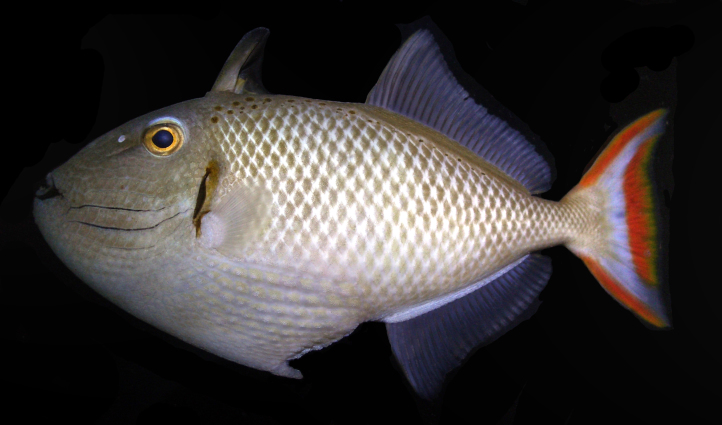


**Table 1.** Proportional measurements and counts of type specimens of *Xanthichthys greenei* sp.n., expressed as a percentage of standard length. Numbers in parentheses represent counts from right side, if different from left side. Cheek groove counts represented as strongly pigmented + weakly pigmented.

	Holotype	Paratypes				
Proportional Measurements	BPBM 40262	BPBM 40228	BPBM 40342	BPBM 40342	USNM (pending)	CAS 236257
Standard Length (SL)	154 mm	139 mm	144 mm	97 mm	113 mm	109 mm
Greatest depth of body	49	45	49	49	47	50
Depth of body at A origin	41	43	42	42	42	42
Width of body	20	20	20	20	19	19
Head length	32	32	31	33	31	31
Snout length	21	21	21	21	20	20
Snout to first dorsal fin	33	32	33	35	33	33
Snout to anal fin	69	67	68	69	67	68
Base of second dorsal fin	36	37	37	35	36	36
Base of anal fin	30	32	31	30	31	31
Eye diameter	6.5	7.2	7.3	8.8	8.4	8.7
Interorbital width	12	12	13	13	12	12
Length of gill opening	10	10	10	9	10	10
Depth of caudal peduncle	8.4	8.6	9.0	8.8	8.4	8.7
Length of first dorsal spine	14	13	17	16	16	16
Length of longest dorsal ray	19	22	19	21	19	20
Length of longest anal ray	18	19	17	19	16	19
Interdorsal space	25	24	25	23	25	24
Length of caudal fin	26	26	24	21	23	24
Caudal concavity	4.5	5.8	5.6	2.1	4.4	3.7
Length of pectoral fin	11	13	13	12	14	12
Counts						
Dorsal-fin spines	3	3	3	3	3	3
Dorsal-fin rays	29	29	29	29	29	29
Anal-fin rays	25	25	25	25	25	25
Pectoral-fin rays	13	13	14 (13)	13	13	13
Body scale rows	34	35	35	34	34	33
Head scale rows	18	17	17	18	17	18
Cheek grooves	3+0	3+1	3+1	3+0	3+1	3+0
Vertebrae	18	18	18	18	18	18

**Table 2.** Comparison of diagnostic characters for *Xanthichthys* species.

Species	Geographic Range	Pigmented Cheek Grooves	Maximum Size	Body Scale Rows
*Xanthichthys greenei*	Kiritimati	3	154 mm	33-35
*Xanthichthys ringens*	Western Atlantic	3	250 mm	39-44
*Xanthichthys lineopunctatus*	W. Indian Ocean; Okinawa	3	260 mm	44-50
*Xanthichthys mento*	Antitropical Pacific; Tropical E. Pacific	5	300 mm	41-50
*Xanthichthys auromarginatus*	W. Indian Ocean to Hawaii	5	227 mm	42-47
*Xanthichthys caeruleolineatus*	W. Indian Ocean to Hawaii	5-6	420 mm	40-48
